# Antibacterial activity of tamoxifen derivatives against methicillin-resistant *Staphylococcus aureus*

**DOI:** 10.3389/fphar.2025.1549288

**Published:** 2025-04-30

**Authors:** Irene Molina Panadero, Javier Falcón Torres, Abdelkrim Hmadcha, Salvatore Princiotto, Luigi Cutarella, Mattia Mori, Sabrina Dallavalle, Michael S. Christodoulou, Younes Smani

**Affiliations:** ^1^ Andalusian Center of Developmental Biology, Consejo Superior de Investigaciones Científicas (CSIC), University of Pablo de Olavide - Seville, Seville, Spain; ^2^ Department of Molecular Biology and Biochemical Engineering, University of Pablo de Olavide, Seville, Spain; ^3^ Biosanitary Research Institute (IIB-VIU), Valencian International University (VIU), Valencia, Spain; ^4^ Department of Food, Environmental and Nutritional Sciences (DeFENS), University of Milan, Milan, Italy; ^5^ Department f Biotechnology, Chemistry and Pharmacy, University of Siena, Siena, Italy

**Keywords:** *Staphylococcus aureus*, tamoxifen derivatives, resistance, infection, treatment

## Abstract

The development of new antimicrobial therapeutic strategies requires urgent attention to prevent the tens of millions of deaths predicted to occur by 2050 as a result of multidrug-resistant (MDR) bacterial infections. This study aimed to discover new tamoxifen derivatives with antimicrobial potential, particularly targeting methicillin-resistant *Staphylococcus aureus* (MRSA). The minimum inhibitory concentration (MIC) of 22 tamoxifen derivatives was determined against *S. aureus* reference and MRSA strains using microdilution assays. The antibacterial effects of selected tamoxifen derivatives against MRSA (USA7 strain) were assessed through bacterial growth assays. Additionally, bacterial membrane permeability and molecular dynamics (MD) simulation assays were performed. The MIC of the tamoxifen derivatives against reference *S. aureus* and MRSA strains ranged from to 16 to >64 μg/mL. Bacterial growth assays demonstrated that tamoxifen derivatives **2**, **5**, and **6**, the only compounds bearing the electron-donating hydroxyl group in the para position on both phenyl rings of the tamoxifen skeleton, dose-dependently reduced the growth of the USA7 strain. Moreover, treatment of MRSA with derivatives **2** and **5** resulted in a slight increase of membrane permeabilization. Extensive MD simulations on the interaction between **5** and **6** and the *S. aureus* membrane model suggest that the compounds do not act by destabilizing the membrane integrity. These findings suggest that tamoxifen derivatives exhibit antibacterial activity against MRSA, potentially broadening the spectrum of available drug treatments for combating antimicrobial-resistant *S. aureus*.

## 1 Introduction

In the last decades, Gram-positive bacteria have demonstrated an increasing antimicrobial resistance, driven by various genomic, transcriptomic, and proteomic adaptations (Yelin and Kishony, 2018). This alarming trend aligns with a concerning decline in the development of new antibiotics, a phenomenon often referred to as the “Post-Antibiotic Era” (Bagley and Outterson, 2017). This situation underscores the urgent need for effective solutions, a concern highlighted by numerous institutions. Consequently, there is a growing demand for innovative antimicrobial therapeutic approaches, including the exploration of non-antibiotic compounds and drug repurposing, both as monotherapy and in combination with the limited clinically relevant antibiotics currently available (Canturri and Smani, 2022).

Among the various strategies employed in the discovery of new antibacterial agents, drug repurposing is one of the most exploited (Canturri and Smani, 2022; Schadich et al., 2022; Ćavar Zeljković et al., 2022; Schadich et al., 2025). A very representative example of such approach is tamoxifen, a well-known chemotherapic drug, widely used for decades as the gold standard for the treatment of estrogen receptor positive breast cancers and related metastatic forms (Green and Carroll, 2007). Recently, tamoxifen has exhibited relevant antibacterial properties against a range of pathogenic microorganisms, including Gram-positive *Staphylococcus epidermidis*, and *Enterococcus faecalis* and Gram-negative *Escherichia coli* and *Acinetobacter baumannii* (Miró-Canturri et al., 2021a; Miró-Canturri et al., 2021b). This antimicrobial effect may be ascribed to the cytochrome P450-mediated metabolism of tamoxifen, resulting in the generation of three major metabolites: *N*-desmethyltamoxifen, 4-hydroxytamoxifen and endoxifen (Miró-Canturri et al., 2021a; Klein et al., 2013).

Only a limited number of studies have reported the activity of these metabolites against various infectious agents (Miró-Canturri et al., 2021a; Miró-Canturri et al., 2021b; Selyunin et al., 2019; Montoya and Krysan, 2018; Weinstock et al., 2019; Buts et al., 2014; Chen et al., 2014). Among these, 4-hydroxytamoxifen has garnered attention for its chemical behavior as a weak base, since it has been observed that such property is responsible for the protection of cells and mice against lethal Shiga toxin 1 (STx1) or Shiga toxin 2 (STx2) toxicosis (Selyunin et al., 2019). It has also demonstrated efficacy against *Plasmodium falciparum* and *Cryptococcus neoformans* (Weinstock et al., 2019; Buts et al., 2014). Furthermore, when used in monotherapy, 4-hydroxytamoxifen has displayed activity against *Mycobacterium tuberculosis* (with a MIC_50_ of approximately 2.5–5 μg/mL) (Chen et al., 2014). Endoxifen’s activity was studied against C. neoformans, revealing a MIC of 4 μg/mL (Buts et al., 2014). The combination of N-desmethyltamoxifen, 4-hydroxytamoxifen and endoxifen exhibited MIC_50_ values of 8 and 16 μg/mL against clinical isolates of *A. baumannii* and *E. coli*, and MIC_50_ values of 1 and 2 μg/mL against clinical isolates of *S. epidermidis* and *E. faecalis* (Miró-Canturri et al., 2021a; Miró-Canturri et al., 2021b).

To identify new chemical compounds with antimicrobial potential, a collection of 22 tamoxifen derivatives, bearing different electron-withdrawing or electron-donating substituents on the aromatic rings A and B (Table 1, supplementary data) was tested against methicillin-resistant *S. aureus* (MRSA).

**TABLE 1:** MIC values of tamoxifen derivatives **1**–**22** for standard reference *S. aureus* strain.

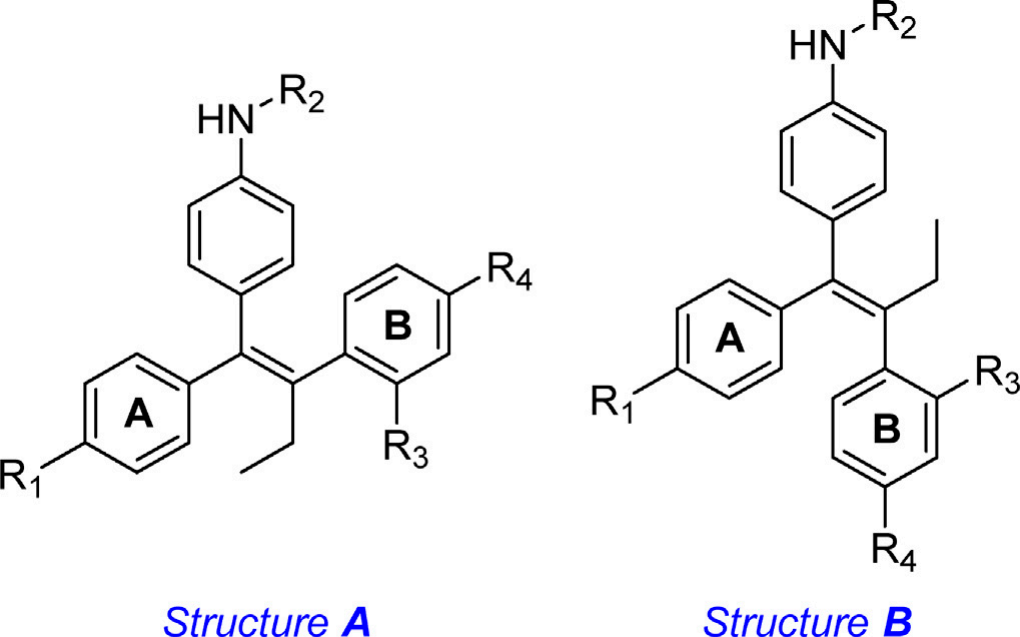							
Compound	Structure	R_1_	R_2_	R_3_	R_4_	*S. aureus* ATCC 1556	
						MIC (μg/mL)	MIC (µM)
**1**	A	H	H	H	H	>64	>214
**2**	A	OH	CON(CH_2_CH_3_)_2_	H	OH	**32**	**74.3**
**3**	A	OBn	COCH_2_CH_3_	H	OBn	>64	>113
**4**	B	OBn	COCH_2_CH_3_	H	OBn	>64	>113
**5**	A	OH	CON(CH_3_)_2_	H	OH	**32**	**79.5**
**6**	B	OH	CON(CH_3_)_2_	H	OH	**16**	**39.8**
**7**	A	OCH_3_	CO*i*Propyl	H	H	>64	>160
**8**	B	OCH_3_	CO*i*Propyl	H	H	>64	>160
**9**	A	H	CO*i*Propyl	H	H	>64	>173
**10**	B	H	CO*i*Propyl	H	H	>64	>173
**11**	B	H	CO*i*Propyl	H	OBn	>64	>134
**12**	A	H	CO*i*Propyl	H	OBn	>64	>134
**13**	A	OH	CO*i*Propyl	Cl	Cl	>64	>141
**14**	B	OH	CO*i*Propyl	Cl	Cl	>64	>141
**15**	A	OCH_3_	CO*i*Propyl	H	OBn	>64	>126
**16**	B	OCH_3_	CO*i*Propyl	H	OBn	>64	>126
**17**	B	OCH_3_	CO*i*Propyl	H	OH	>64	>154
**18**	A	OCH_3_	CO*i*Propyl	H	OH	>64	>154
**19**	A	OH	CO*i*Propyl	H	H	>64	>166
**20**	B	OH	CO*i*Propyl	H	H	>64	>166
**21**	A	OBn	CO*i*Propyl	H	OBn	>64	>110
**22**	B	OBn	CO*i*Propyl	H	OBn	>64	>110

## 2 Materials and methods

### 2.1 Bacterial strains

A total of 7 MRSA clinical isolates and 1 *S. aureus* reference ATCC 1556 strain were used in this study (Docobo Pérez, 2009).

### 2.2 Antimicrobial agent and tamoxifen derivatives

The small library of tamoxifen derivatives was prepared as described in literature, starting from appropriately substituted benzophenones which underwent McMurry olefination (Christodoulou et al., 2013; Christodoulou et al., 2016). The structures of the tested compounds are presented in Table 1.

### 2.3 *In vitro* susceptibility testing

The MICs of tamoxifen derivatives (from 16 to 64 μg/mL and from 39.8 to 214 μM) were determined against reference and MRSA strains in two independent experiments using the broth microdilution method, in accordance with the standard guidelines of the European Committee on Antimicrobial Susceptibility Testing (EUCAST) (European Committee on Antimicrobial Susceptibility Testing, 2022). A 5 × 10^5^ cfu/mL inoculum of each strain was cultured in Luria Bertani (LB) and added to U bottom microtiter plates (Deltlab, Spain) containing the studied tamoxifen derivatives. The plates were incubated for 18 h at 37°C. *Pseudomonas aeruginosa* ATCC 27853 was used as the positive control strain.

### 2.4 Bacterial growth curves

To determine the antibacterial and synergistic effects, duplicate bacterial growth curves were performed for MRSA USA7 strain. A 1/200 dilution of an overnight bacterial cultures grown in LB at 37°C with continuous agitation at 180 rpm was performed in LB in 96-well plate in the presence of 1×, 2× and 4× MIC of compounds **2**, **5** and **6**. A drug-free broth was evaluated in parallel as control. Absorbance measurements at 600 nm every 20 min for 24 h were conducted using a Tecan spectrophotometer (model XYZ-2000, Austria).

### 2.5 Membrane permeability assays

The bacterial cells were grown in LB broth and incubated in the absence or presence of 1) 1x MIC of compounds **2**, **5** and **6** for 3 h. The pellet was harvested by ultracentrifugation at 4600 g for 15 min. The bacterial cells were washed with PBS 1X, and after centrifugation in the same condition described before, the pellet was resuspended in 100 µL of PBS 1X containing 10 μL of Ethidium Homodimer-1 (ThermoFisher, United States). After 10 min of incubation, 100 μL was placed into a 96-well plate to measure fluorescence for 300 min using a Typhoon FLA 9000 laser scanner (GE Healthcare Life Sciences, United States) and quantified using ImageQuant TL software (GE Healthcare Life Sciences, United States) (Miró-Canturri et al., 2020).

### 2.6 Molecular dynamics simulations assays

The symmetric lipid bilayer membrane of *S. aureus* was built using the CHARMM-GUI Membrane Builder Tool (Li et al., 2021; Jo et al., 2008). This system was designed to mimic the phospholipid composition of the *S. aureus* membrane, which consists of 56.8% phosphatidylglycerol (PG), 37.9% Lys-PG, and 5.3% diphosphatidylglycerol (DPG), also referred as Cardiolipin (CL), in agreement with previous molecular dynamics (MD) studies (Kim et al., 2018; Princiotto et al., 2024; Witzke et al., 2016). Each lipid bilayer contained a total of 95 lipid molecules, positioned with their centers at z = 0., surrounded by a water layer with a thickness of 50 Å. The system was neutralized using Na+ and Cl-ions at a concentration of 0.145 M, as suggested by CHARMM-GUI.

Derivatives **5** and **6** were drawn using the Sketchpad powered by Marvin JS included in the ligand reader and modeler module of CHARMM-GUI, and were parameterized using the standard CHARMM force field (FF) (Kim et al., 2017). The Multicomponent Assembler tool was used to randomly distribute the small molecules under investigation within the solvent area. The membrane systems containing these small molecules were then parameterized with a membrane thickness of 50 Å and a box XY length of 79.50 Å (Huang et al., 2016; Lee et al., 2020). Following system construction with CHARMM-GUI, the topology and coordinate files were generated for AMBER (Lee et al., 2016; Brooks et al., 2009).

To investigate the interaction between **5** and **6** with the *S. aureus* membrane model, MD simulations were conducted using AMBER22 (Salomon-Ferrer et al., 2013; Case et al., 2005). The initial system was energy minimized for a total of 40000 steps., with the first 1500 steps employing the steepest descent algorithm, followed by the conjugate gradient algorithm for the remaining steps. A non-bonded cut-off of 10Å was used. After energy minimization, each system was gradually heated to 300 K over 900 ps at constant volume using the Langevin thermostat with a collision frequency of 2 ps-1, and then left at 300 K at constant volume for 200 ps. Box density was equilibrated at constant pressure and constant temperature (300 K) over 1 ns using the Berendsen barostat.

Following density equilibration, a preliminary 50 ns MD simulation was conducted at constant pressure. Subsequently, MD trajectories were generated for 500 ns; two independent replicates for each compound were run In all MD simulations, no positional restraints were applied.

Analysis of MD trajectories was performed using the CPPTRAJ (Roe and Cheatham, 2013) program from the AmberTools package. This analysis included calculation of the mass densities of derivatives within the system along the z-axis. Small molecules interactions with the membrane were visually inspected with PyMol (Delano, 2002).

### 2.7 Statistical analysis

Group data are presented as means ± standard errors of the means (SEM). The one-way ANOVA test was used to determine differences between means using the GraphPad Prism 9. A *p*-value <0.05 was considered significant.

## 3 Results

### 3.1 Antimicrobial activity of tamoxifen derivatives

Twenty-two tamoxifen derivatives (Table 1) were subjected to evaluation to determine their MIC against *S. aureus* ATCC 1556 reference strain. Only the tamoxifen derivatives **2**, **5** and **6** showed MIC for *S. aureus* ATCC 1556 strain ranging from 16 to 32 μg/mL (39.8–79.5 μM), while the rest of the compounds did not show MIC values < 64 μg/mL (110–214 μM) (Table 1).

A comparison between the biological results and the chemical structures of the tamoxifen derivatives allowed some interesting structure-activity relationships outcomes. In particular, the most active compounds **2**, **5** and **6** share the presence of the hydroxyl group in para position on both phenyl rings, A and B. All the derivatives bearing the hydroxyl group on only one of the phenyl rings resulted inactive (compounds **13**, **14**, **17**, **18**, **19** and **20**). In the case of no substituents on the phenyl rings (compounds **1**, **9** and **10**) or in presence of electron-donating substituents diverse from the hydroxyl group on one or both phenyl rings A and B, the derivatives resulted inactive as well (compounds **3**, **4**, **7**, **8**, **11**, **12**, **15**, **16**, **21** and **22**).

The most promising derivatives were selected on the base of a MIC ≤64 μg/mL and were evaluated against 7 clinical isolates of MRSA to extend their biological evaluation to the antimicrobial effect on resistant strains. As shown in Table 2, the MICs of tamoxifen derivatives **2**, **5** and **6** for MRSA strains ranged from 16 to 64 μg/mL.

**TABLE 2:** MIC values of tamoxifen derivatives **2**, **5,** and **6** against methicillin-resistant *S. aureus* clinical isolates.

Strain	2		5		6	
	MIC (μg/mL)	MIC (µM)	MIC (μg/mL)	MIC (µM)	MIC (μg/mL)	MIC (µM)
USA1	32	74.3	32	79.5	16	39.8
USA2	32	74.3	64	159	16	39.8
USA3	32	74.3	64	159	16	39.8
USA4	32	74.3	64	159	16	39.8
USA5	64	148	64	159	16	39.8
USA6	64	148	64	159	16	39.8
USA7	32	74.3	64	159	16	39.8

### 3.2 Time dependent antibacterial effects

**FIGURE 1:**
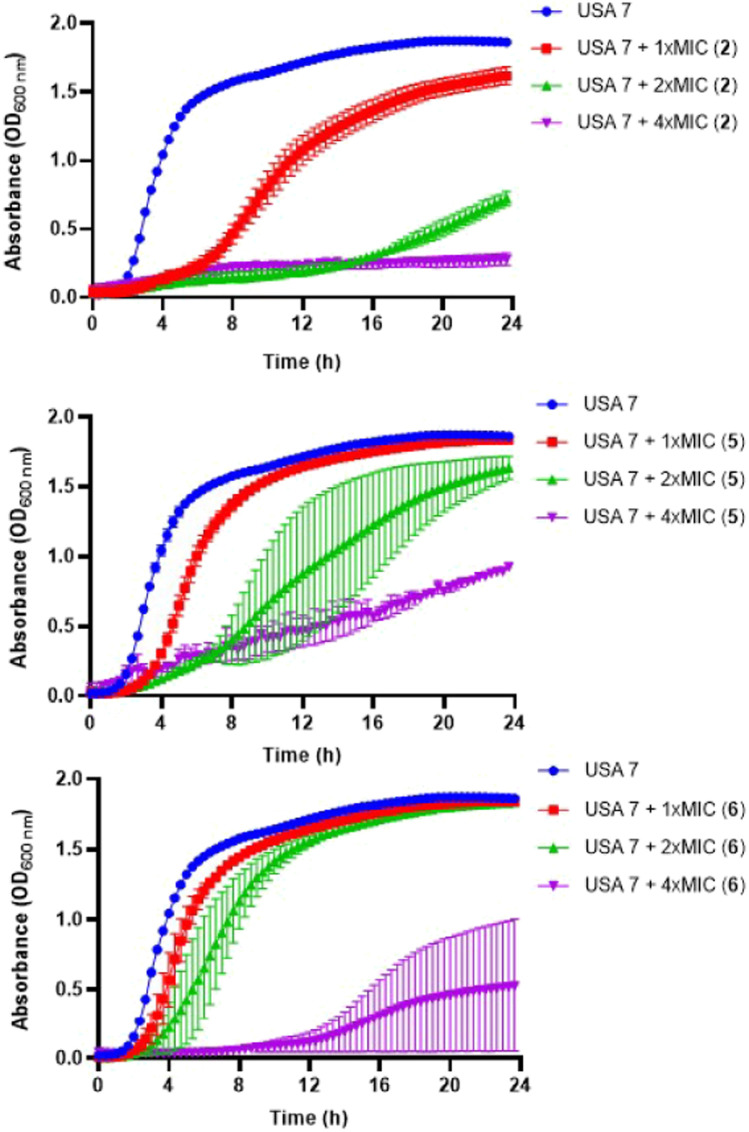
Antibacterial activity of tamoxifen derivatives against clinical methicillin-resistant *S. aureus*. Bacterial growth curves of MRSA USA7 strain in the presence of 1×, 2× and 4× MIC of tamoxifen derivative **2, 5 or 6** for 24 h. Data are represented as mean from two independent. Replicates and experiments. COL: colistin.

Using bacterial growth, we examined the antibacterial activity of the selected tamoxifen derivatives against MRSA USA7 strain. Compounds **2**, **5** and **6** at concentrations of 1×, 2× and 4× MIC, reduced the growth of this strain in a concentration-dependent manner during 24 h. The inhibitory effects of tamoxifen derivatives **2** and **5** at 1×, 2× and 4× MIC on bacterial were more pronounced and began earlier than with tamoxifen derivative **6** (Figure 1).

### 3.3 Effect of tamoxifen derivatives on the bacterial cell membrane

In order to determine the mode of action of the selected tamoxifen derivatives, their effect on the membrane permeability of USA7 strain was evaluated by incubation with ethidium homodimer-1, a fluorescent marker known to enter bacterial cells when the membrane integrity is compromised.

**FIGURE 2:**
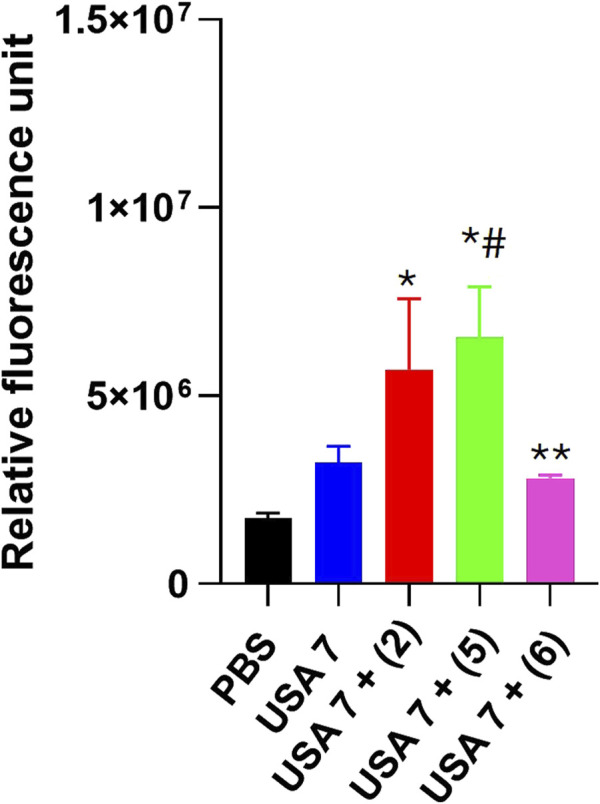
Effect of tamoxifen derivatives on the bacterial permeability against clinical methicillin-resistant *S. aureus*. The membrane permeabilization of MRSA USA7 strain in the presence of 1xMIC of tamoxifen derivative **2**, **5** or **6**, incubated for 10 min, was quantified by Typhon Scanner. Data are represented as mean ± SEM from three independent replicates and experiments. *P < 0.05 vs. PBS, #*P* < 0.05 vs. USA7, *P < 0.05 vs. USA7 + (5).

Fluorescence monitoring using a Typhoon FLA scanner for 3 h showed a slight increase in the cellular fluorescence of the USA7 strain when treated with sub-MIC of the tamoxifen derivatives **2** and **5**, but not for **6**. (Figure 2).

### 3.4 Tamoxifen derivatives interaction with bacterial membrane

To investigate the interaction between tamoxifen derivates **5** and **6** and the bacterial membrane model, and to assess how subtle structural modifications (i.e., *cis/trans* conformation) might affect their ability to embed within the target, unbiased MD simulations were carried out starting from the small molecules being placed in the solvent area (Li et al., 2021; Jo et al., 2008; Piggot et al., 2011; Kim et al., 2018; Princiotto et al., 2024; Witzke et al., 2016; Joodaki et al., 2022).

**FIGURE 3:**
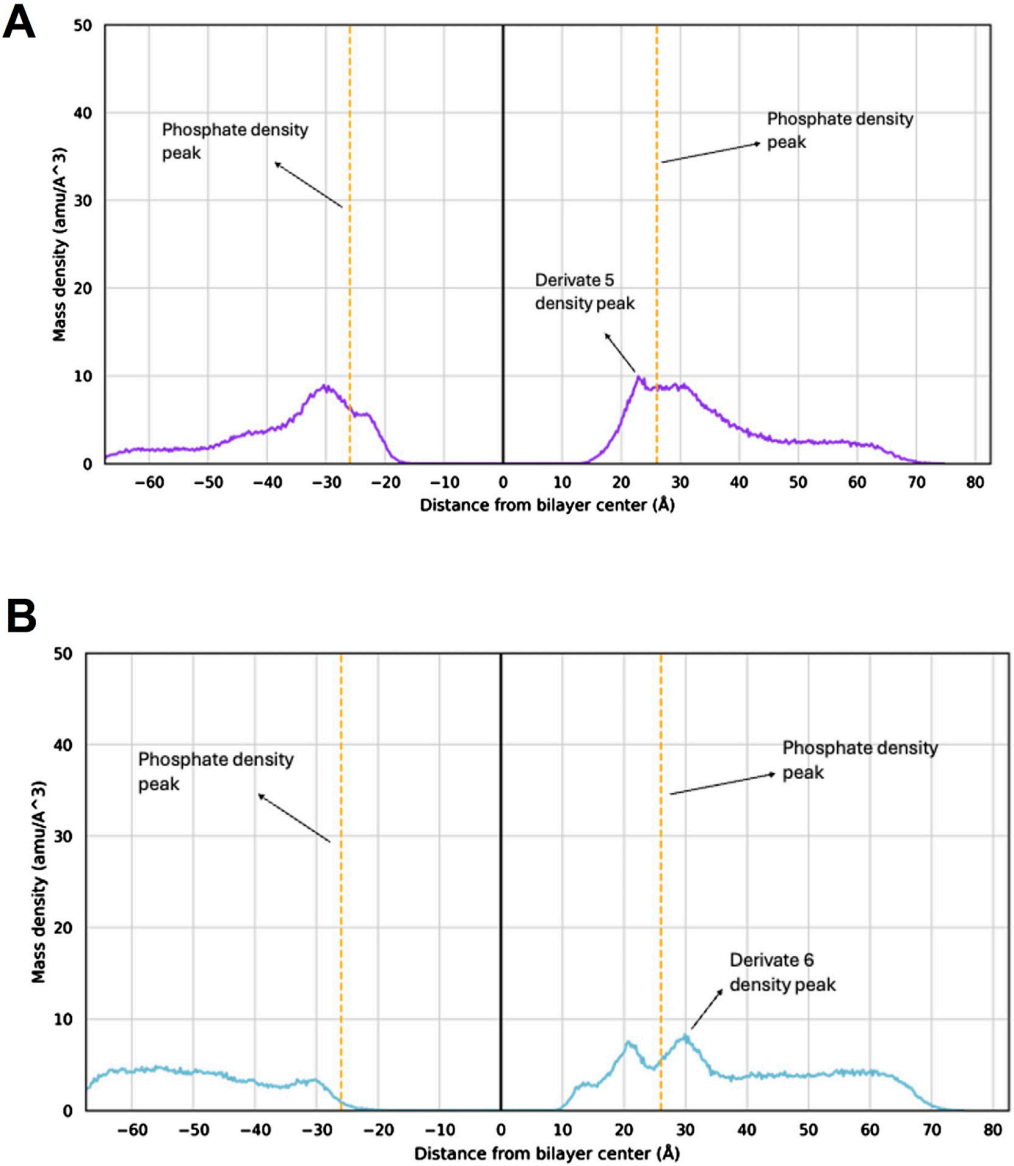
Mass density profile of 5 and 6 within the *S. aureus* membrane model. Mass density profile of 5 **(A)** and 6 **(B)** with the corresponding density peaks highlighted. The density peaks of the lipid phosphate groups are shown as orange dashed lines, with maximum density at −26 Å and +26 Å. The mass center of the membrane model is at point 0. The molecules’ mass density peaks indicate the position of each compound within the bilayer system as extracted from the MD simulations.

Different from previous findings with different scaffolds (Princiotto et al., 2024), for derivatives **5** and **6** the density plots clearly evidence that the molecules are unable to penetrate deeply into the membrane model (Figures 3A,B). In fact, density peaks of the two derivatives slightly exceed the density peak of the phosphates that represent the membrane outermost layer. These results suggest that the *S. aureus* membrane might not be the molecular target of **5** and **6** antibacterial efficacy.

**FIGURE 4:**
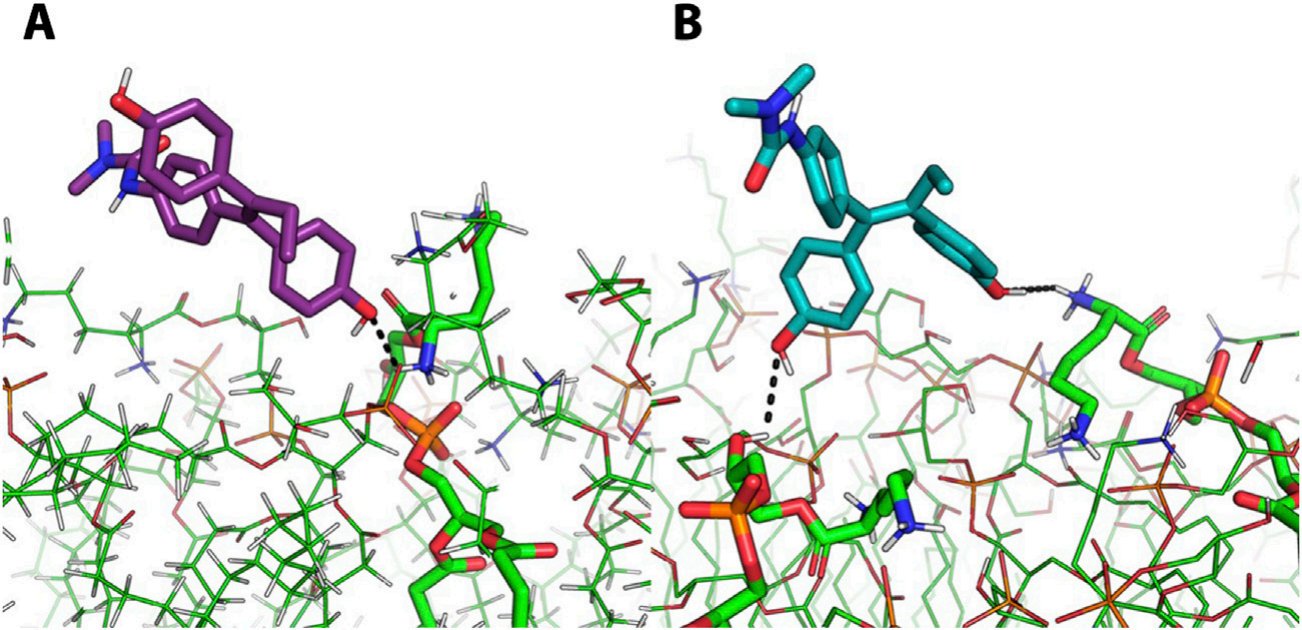
Representative of the most representative frame of MD simulations of molecules 5 and 6. Representative MD frames corresponding to the minimum distances between derivate **5** (A, purple sticks) or derivate **6** (B, light blue sticks) and the *S. aureus* membrane model. The interactions between these two derivates and the NH3^+^ moiety **(A,B)** and -OH moiety **(B)** of phosphatidylglycerol residues is represented by black dashed lines.

To further provide atomistic details of this outcome, the distance between each molecule and the lipid bilayer was monitored along MD trajectories. Then, the MD frame corresponding to the smallest intermolecular distance was extracted and visually inspected. A labile H-bond interaction between the phenol moiety of the molecules and the protonated ammino group (i.e., R-NH_3_^+^) of a phosphatidylglycerol residues at the membrane interface with the solvent was observed (Figures 4A,B). Unlike **5**, derivative **6** also establishes an additional interaction with the -OH group of a phosphatidylglycerol residue (Figure 4B). However, this interaction is not stable in time and failed to promote molecules embedding into the *S. aureus* membrane model within the simulation time, which further reinforce the hypothesis that **5** and **6** exploit their antibacterial potentials through the interaction with target that differ from the bacterial membrane.

## 4 Discussion

The rise of multidrug resistant *S. aureus* has led to a wide use of the last options for the treatment of severe infections caused by this microorganism, and to the consequent acquired antimicrobial resistance worldwide in the last decades (Abebe and Birhanu, 2023). In previous studies, tamoxifen and its metabolites garnered attention as a potential repurposed drug for treatment of infectious diseases (Montoya and Krysan, 2018).

Due to the antibacterial effect of tamoxifen and its metabolites *N*-desmethyltamoxifen, 4-hydroxytamoxifen and endoxifen against *S. epidermidis* and *A. baumannii* (Miró-Canturri et al., 2021a; Miró-Canturri et al., 2021b), we hypothesized that tamoxifen derivatives may enhance this antibacterial activity against MRSA. For this purpose, twenty-two tamoxifen derivatives were tested against *S. aureus* reference strain.

Three tamoxifen derivatives **2**, **5** and **6** in monotherapy showed antibacterial activity against MRSA strains being the MIC between 16 and 64 μg/mL, which overall fell within the range of other known antibiotics such as amikacin, teicoplanin, sulfonamides nitrofurantoin (European Committee on Antimicrobial Susceptibility Testing, 2024; CLSI, 2024). However, none of the rest of tested tamoxifen derivatives exhibited activity against MRSA. Reasons for this difference could be related to the chemical structure of these derivatives, being **2**, **5** and **6** the only compounds bearing the electron-donating hydroxyl group in para position on both phenyl rings A and B.

The antibacterial activity of tamoxifen derivatives **2** and **5** at 1x, 2x and 4x MIC against MRSA USA7 strain was more pronounced and began earlier than with tamoxifen derivative **6**. This result may be related to the *trans* stereochemistry (structure A) of compounds **2** and **5** and their ability to act on the plasma membrane permeability of *S. aureus*, as observed with tamoxifen metabolites against Gram-negative bacteria (Miró-Canturri et al., 2021a). Membrane permeability assays indicate that tamoxifen derivatives **2** and **5**, but not **6**, produced a slight increase in membrane permeability of MRSA. In support of this hypothesis, tamoxifen has been shown to increase the membrane permeability of *Streptococcus pneumoniae* by perturbing the phospholipid bilayer (Ortiz-Miravalles et al., 2023). Notably, MD simulations were carried out to simulate atomistic details of the interaction between **5** and **6** with the *S. aureus* membrane model, showing that, although an interaction does occur, the membrane integrity is not perturbed by the compounds.

As such, the *S. aureus* membrane might not be the molecular target of these derivatives. Alternative mechanisms of action, rather than the perturbation of the plasma membrane, cannot be ruled out. Of note, in other microorganisms such as fungi, the mechanism of action of the tamoxifen is well-documented and involves its binding to calmodulin (Dolan et al., 2009; Butts et al., 2014). Moreover, the tamoxifen metabolite, 4-hydroxytamoxifen, can potentially inhibit bacterial phospholipase D in *P. aeruginosa* (Scott et al., 2015). Additional research focusing on understanding the mechanism of action of the tested tamoxifen derivatives against MRSA would be of significant interest since different chemical structures resulted active on the aforementioned strain, thus providing a better therapeutic efficacy.

The antimicrobial activity of the selected derivatives identified in this work hints at a promising potential that deserves to be further explored *in vivo* after determining their pharmacokinetic parameters. However, *in vitro* bacterial growth showed a progressive regrowth of the MRSA USA7 strain after treatment with these tamoxifen derivatives, suggesting that acquired resistance could take place. Nevertheless, it should be noted that the MICs of tamoxifen derivatives **2**, **5**, and **6** against the USA7 strain in this bacterial growth condition are 32, 32, and 16 μg/mL, respectively. These values are lower than the concentrations originally tested, which were set at 2x and 4 MIC for those derivatives. In other words, the growth of the USA7 strain is inhibited at lower concentrations than expected based on the initial MIC, suggesting that tamoxifen derivatives **2**, **5**, and **6** are more effective under these specific growth conditions. Further investigations, including the determination of tamoxifen derivatives concentration during the bacterial growth assay, are necessary to better understand the regrowth of this strain in their presence. In addition, elucidating the mechanism of action of these derivatives and determining their optimal dosage could ensure therapeutic effectiveness in treating severe infections caused by multidrug-resistant *S. aureus*.

Finally, the prospect of repurposing tamoxifen derivatives as antimicrobial agents introduces a significant challenge: the need to dissociate their antimicrobial properties from tamoxifen’s original anti-estrogen activity. To achieve this, any tamoxifen derivative intended for use as an antimicrobial must be designed or modified to lose its capacity to interact with estrogen receptors while preserving its antimicrobial potency.

## 5 Conclusion

The findings of this study provide new insights into the use of a diverse group of tamoxifen derivatives against MRSA, a pathogen for which treatment options are severely restricted. We demonstrated that tamoxifen derivatives exhibit *in vitro* antibacterial activity against both reference strain and clinical isolates of *S. aureus*, without targeting the bacterial membrane. Moreover, their activity correlates with the presence of a hydroxyl group in the para position on both derivative phenyl rings.

## Data Availability

The original contributions presented in the study are publicly available. This data can be found here: https://zenodo.org/records/14965780.
