# Supplementary material for: Antibacterial activity of tamoxifen derivatives against methicillin-resistant Staphylococcus aureus
Source: Front Pharmacol. 2025 Apr 30;16:1549288. doi: 10.3389/fphar.2025.1549288 (PMC12075203; doi:10.3389/fphar.2025.1549288)
Supplement: Supplementary file 1 [file DataSheet1.docx]

**Antibacterial activity of tamoxifen derivatives against methicillin-resistant *Staphylococcus aureus***

Irene Molina Panadero^1^, Javier Falcón Torres,^1^ Karim Hmadcha,^2,3^ Salvatore Princiotto,^4^ Luigi Cutarella,^5^ Mattia Mori,^5^ Sabrina Dallavalle,^4^ Michael S. Christodoulou,^4*^ and Younes Smani^1,2*^

^1^Andalusian Center of Developmental Biology, CSIC, University of Pablo de Olavide - Seville (Spain).

^2^Department of Molecular Biology and Biochemical Engineering, University of Pablo de Olavide, Seville (Spain).

^3^Biosanitary Research Institute (IIB-VIU), Valencian International University (VIU), Valencia, Spain.

^4^Department of Food, Environmental and Nutritional Sciences (DeFENS), University of Milan, via Celoria 2, 20133 Milan, Italy.

^5^Department f Biotechnology, Chemistry and Pharmacy, University of Siena, via Aldo Moro 2, 53100 Siena, Italy.

**Table of contents:**

1. ^1^H NMR and ^13^C spectrums of compound **1 S2**
2. ^1^H NMR and ^13^C spectrums of compound **2 S2**
3. ^1^H NMR and ^13^C spectrums of compound **3 S3**
4. ^1^H NMR and ^13^C spectrums of compound **4 S3**
5. ^1^H NMR and ^13^C spectrums of compound **5 S4**
6. ^1^H NMR and ^13^C spectrums of compound **6 S4**
7. ^1^H NMR and ^13^C spectrums of compound **7 S5**
8. ^1^H NMR and ^13^C spectrums of compound **8 S5**
9. ^1^H NMR and ^13^C spectrums of compound **9 S6**
10. ^1^H NMR and ^13^C spectrums of compound **10 S6**
11. ^1^H NMR and ^13^C spectrums of compound **11 S7**
12. ^1^H NMR and ^13^C spectrums of compound **12 S7**
13. ^1^H NMR and ^13^C spectrums of compound **13 S8**
14. ^1^H NMR and ^13^C spectrums of compound **14 S8**
15. ^1^H NMR and ^13^C spectrums of compound **15 S9**
16. ^1^H NMR and ^13^C spectrums of compound **16 S9**
17. ^1^H NMR and ^13^C spectrums of compound **17 S10**
18. ^1^H NMR and ^13^C spectrums of compound **18 S10**
19. ^1^H NMR and ^13^C spectrums of compound **19 S11**
20. ^1^H NMR and ^13^C spectrums of compound **20 S11**
21. ^1^H NMR and ^13^C spectrums of compound **21 S12**
22. ^1^H NMR and ^13^C spectrums of compound **22 S12**

**Compound 1: (*Z*)-4-(1,2-diphenylbut-1-en-1-yl)aniline**

^1^H NMR (CDCl_3_): *δ* = 7.37 (2H, t, *J* 7.2 Hz, H-3’’’), 7.31 – 7.27 (3H, m, H-2’’’ and H-4’’’), 7.23 – 7.13 (5H, m, H-2’’, H-3’’ and H-4’’), 6.69 (2H, d, *J* 8.8 Hz, H-3), 6.40 (2H, d, *J* 8.8 Hz, H-2), 2.48 (2H, q, *J* 7.6 Hz, C*H*_2_CH_3_), 0.950 (3H, t, *J* 7.6 Hz, CH_2_C*H*_3_); ^13^C NMR (CDCl_3_): *δ* = 144.7 (C-1’’’), 144.0 (C-1), 143.5 (C-1’’), 141.4 (C-2’), 139.4 (C-1’), 134.7 (C-4), 132.3 (C-3), 130.4 (C-2’’), 130.1 (C-2’’’), 128.6 (C-3’’’), 128.4 (C-3’’), 127.0 (C-4’’’), 126.5 (C-4’’), 115.2 (C-2), 29.55 (*C*H_2_CH_3_), 13.99 (CH_2_*C*H_3_).

**Compound 2: (*E*)-3-(4’-(1’’,2’’-bis(4’’’,4’’’-hydroxyphenyl)but-1’’-enyl)phenyl)-1,1-diethylurea**

^1^H NMR (acetone-d_6_, 600 MHz): *δ* = 7.41 (1Η, s, N*H*), 7.21 (2H, d, *J* 8.4 Hz, H-2’), 7.05 (2H, d, *J* 8.4 Hz, H-2’’’), 6.96 (2H, d, *J* 8.4 Hz, H-2’’’’), 6.83 (2H, d, *J* 8.4 Hz, H-3’’’), 6.73 (2H, d, *J* 8.4 Hz, H-3’), 6.65 (2H, d, *J* 8.4 Hz, H-3’’’’), 3.36 (4H, q, *J* 7.2 Hz, N(C*H*_2_CH_3_)_2_), 2.45 (2H, q, *J* 7.2 Hz, C*H*_2_CH_3_), 1.11 (6H, t, *J* 7.2 Hz, N(CH_2_C*H*_3_)_2_), 0.911 (3H, t, *J* 7.2 Hz, CH_2_C*H*_3_); ^13^C NMR (acetone-d_6_, 50 MHz): *δ* = 156.1 (CO), 155.6 (C-4’’’), 154.3 (C-4’’’’), 140.3 (C-2’’), 138.3 (C-4’), 137.8 (C-1’’), 137.4 (C-1’), 135.3 (C-1’’’), 133.6 (C-1’’’’), 130.7 (C-3’), 130.6 (C-2’’’’), 130.4 (C-2’’’), 118.4 (C-2’), 114.7 (C-3’’’ and C-3’’’’), 40.9 (Ν(*C*H_2_CH_3_)_2_), 28.9 (*C*H_2_CH_3_ from HSQC), 13.4 (Ν(CH_2_*C*H_3_)_2_), 13.1 (CH_2_*C*H_3_).

**Compound 3: (*E*)-*N*-(4-(1’,2’-bis(4’’,4’’’-(benzyloxy) phenyl)but-1’-enyl)phenyl) propionamide**

^1^H NMR (CDCl_3_, 600 MHz): *δ* = 7.46 – 7.31 (10H, m, Ph*H* and Ph*Η*’), 7.18 (2H, d, *J* 8.4 Hz, H-2), 7.13 (2H, d, *J* 8.4 Hz, H-2’’), 7.02 (2H, d, *J* 8.4 Hz, H-2’’’), 7.00 (1H, s, N*H*), 6.95 (2H, d, *J* 8.4 Hz, H-3’’), 6.83 (2H, d, *J* 8.4 Hz, H-3), 6.78 (2H, d, *J* 8.4 Hz, H-3’’’), 5.07 (2H, s, C4’’-OC*H*_2_), 4.99 (2H, s, C4’’’-OC*H*_2_), 2.47 (2H, q, *J* 7.2 Hz, C*H*_2_CH_3_), 2.32 (2H, q, *J* 7.2 Hz, COC*H*_2_CH_3_), 1.20 (3H, t, *J* 7.2 Hz, COCH_2_C*H*_3_), 0.940 (3H, t, *J* 7.2 Hz, CH_2_C*H*_3_); ^13^C NMR (CDCl_3_, 50 MHz): *δ* = 171.7 (CO), 157.6 (C-4’’), 157.2 (C-4’’’), 141.3 (C-2’), 139.6 (C-4), 137.3 (C-1), 137.2 (Ph*C*CH_2_O and Ph*C’*CH_2_O), 136.4 (C-1’), 136.0 (C-1’’), 135.4 (C-1’’’), 131.5 (C-3), 130.8 (C-2’’’), 130.7 (C-2’’), 128.6 (Ph*C’*-ortho), 128.5 (Ph*C*-ortho), 128.0 (Ph*C’*-para), 127.9 (Ph*C*-para), 127.6 (Ph*C’*-meta), 127.5 (Ph*C*-meta), 118.6 (C-2), 114.4 (C-3’’), 114.3 (C-3’’’), 70.1 (C4’’-O*C*H_2_), 70.0 (C4’’’-O*C*H_2_), 30.8 (CO*C*H_2_CH_3_), 29.7 (*C*H_2_CH_3_), 13.7 (CH_2_*C*H_3_), 9.7 (COCH_2_*C*H_3_).

**Compound 4: (*Z*)-*N*-(4-(1’,2’-bis(4’’,4’’’-(benzyloxy) phenyl)but-1’-enyl)phenyl) propionamide**

^1^H NMR (CDCl_3_, 600 MHz): *δ* = 7.52 (2H, d, *J* 8.4 Hz, H-2), 7.43 – 7.29 (10H, m, Ph*H* and Ph*Η*’), 7.18 (3H, m, H-3 and Ν*Η*), 7.04 (2H, d, *J* 8.4 Hz, H-2’’’), 6.80 (2H, d, *J* 8.4 Hz, H-3’’’), 6.78 (2H, d, *J* 8.4 Hz, H-2’’), 6.64 (2H, d, *J* 8.4 Hz, H-3’’), 5.01 (2H, s, C4’’’-OC*H*_2_), 4.93 (2H, s, C4’’-OC*H*_2_), 2.45 (2H, q, *J* 7.2 Hz, C*H*_2_CH_3_), 2.40 (2H, q, *J* 7.2 Hz, COC*H*_2_CH_3_), 1.26 (3H, t, *J* 7.2 Hz, COCH_2_C*H*_3_), 0.928 (3H, t, *J* 7.2 Hz, CH_2_C*H*_3_); ^13^C NMR (CDCl_3_, 50 MHz): *δ* = 171.9 (CO), 157.1 (C-4’’), 156.8 (C-4’’’), 141.0 (C-2’), 140.0 (C-4), 137.2 (Ph*C*CH_2_O and Ph*C’*CH_2_O), 136.4 (C-1’), 136.0 (C-1’’), 134.9 (C-1’’’), 132.0 (C-2’’), 130.6 (C-2’’’), 130.1 (C-3), 129.9 (C-1), 128.5 (Ph*C’*-ortho and Ph*C*-ortho), 127.9 (Ph*C’*-para and Ph*C*-para), 127.5 (Ph*C’*-meta and Ph*C*-meta), 119.4 (C-2), 114.3 (C-3’’’), 113.8 (C-3’’), 69.9 (C4’’’-O*C*H_2_), 69.9 (C4’’-O*C*H_2_), 30.8 (CO*C*H_2_CH_3_), 28.9 (*C*H_2_CH_3_), 13.7 (CH_2_*C*H_3_), 9.7 (COCH_2_*C*H_3_).

**Compound 5: (*E*)-3-(4’-(1’’,2’’-bis(4’’’,4’’’-hydroxyphenyl)but-1’’-enyl)phenyl)-1,1-dimethylurea**

^1^H NMR (CD_3_OD-d_4_, 400 MHz): *δ* = 7.03 (2H, d, *J* 8.4 Hz, H-2’), 7.02 (2H, d, *J* 8.4 Hz, H-2’’’), 6.94 (2H, d, *J* 8.4 Hz, H-2’’’’), 6.78 (2H, d, *J* 8.4 Hz, H-3’’’), 6.76 (2H, d, *J* 8.4 Hz, H-3’), 6.58 (2H, d, *J* 8.4 Hz, H-3’’’’), 2.98 (6Η, s, Ν(C*H*_3_)_2_), 2.46 (2H, q, *J* 7.2 Hz, C*H*_2_CH_3_), 0.930 (3H, t, *J* 7.2 Hz, CH_2_C*H*_3_); ^13^C NMR (CD_3_OD-d_4_, 50 MHz): *δ* = 157.5 (CO), 155.3 (C-4’’’), 154.8 (C-4’’’’), 140.8 (C-2’’), 138.8 (C-4’), 137.6 (C-1’’), 136.9 (C-1’), 135.2 (C-1’’’), 133.5 (C-1’’’’), 130.7 (C-3’), 130.5 (C-2’’’’), 130.2 (C-2’’’), 119.8 (C-2’), 114.4 (C-3’’’), 114.3 (C-3’’’’), 35.3 (Ν(*C*H_3_)_2_), 28.4 (*C*H_2_CH_3_), 12.6 (CH_2_*C*H_3_).

**Compound 6: (*E*)-3-(4’-(1’’,2’’-bis(4’’’,4’’’-hydroxyphenyl)but-1’’-enyl)phenyl)-1,1-dimethylurea**

^1^H NMR (CD_3_OD-d_4_, 400 MHz): *δ* = 7.35 (2H, d, *J* 8.4 Hz, H-2’), 7.11 (2H, d, *J* 8.4 Hz, H-3’), 6.94 (2H, d, *J* 8.4 Hz, H-2’’’’), 6.68 (2H, d, *J* 8.4 Hz, H-2’’’), 6.61 (2H, d, *J* 8.4 Hz, H-3’’’’), 6.44 (2H, d, *J* 8.4 Hz, H-3’’’), 3.05 (6Η, s, Ν(C*H*_3_)_2_), 2.46 (2H, q, *J* 7.2 Hz, C*H*_2_CH_3_), 0.930 (3H, t, *J* 7.2 Hz, CH_2_C*H*_3_); ^13^C NMR (CD_3_OD-d_4_, 50 MHz): *δ* = 157.7 (CO), 155.7 (C-4’’’), 155.2 (C-4’’’’), 140.4 (C-2’’), 137.2 (C-1’), 136.8 (C-1’’), 135.2 (C-4’), 133.5 (C-1’’’), 131.4 (C-1’’’’), 130.7 (C-2’’’), 130.6 (C-2’’’’), 130.3 (C-3’), 119.7 (C-2’), 114.4 (C-3’’’’), 114.3 (C-3’’’), 35.3 (Ν(*C*H_3_)_2_), 28.4 (*C*H_2_CH_3_), 12.6 (CH_2_*C*H_3_).

**Compound 7: (*E*)-*N*-(4-(1-(4-methoxyphenyl)-2-phenylbut-1-en-1-yl)phenyl)isobutyramide**

^1^H NMR (CDCl_3_): *δ* = 7.28 – 7.21 (9H, m, H-2, H-2’’, H-2’’’, H-3’’’ and H-4’’’), 7.04 (1H, br. s, NH), 6.90 (2H, d, *J* 8.4 Hz, H-3’’), 6.83 (2H, d, *J* 8.0 Hz, H-3), 3.85 (3H, s, OCH_3_), 2.52 (2H, q, *J* 7.2 Hz, C*H*_2_CH_3_), 2.47 – 2.40 (1H, m, C*H*(CH_3_)_2_), 1.21 (6H, d, *J* 6.8 Hz, CH(C*H*_3_)_2_), 0.960 (3H, t, *J* 7.2 Hz, CH_2_C*H*_3_); ^13^C NMR (CDCl_3_): *δ* = 175.4 (CO), 159.2 (C-4’’), 143.2 (C-1’’’), 142.6 (C-2’), 140.1 (C-4), 138.7 (C-1’), 136.7 (C-1’’), 136.4 (C-1), 131.9 (C-3), 131.2, 130.3 and 128.4 (C-2’’, C-2’’’ or C-3’’’), 126.6 (C-4’’’), 119.5 (C-2), 114.4 (C-3’’), 55.84 (OCH_3_), 37.21 (*C*H(CH_3_)_2_), 29.61 (*C*H_2_CH_3_), 20.05 (CH(*C*H_3_)_2_), 13.94 (CH_2_*C*H_3_).

**Compound 8: (*Z*)-*N*-(4-(1-(4-methoxyphenyl)-2-phenylbut-1-en-1-yl)phenyl)isobutyramide**

^1^H NMR (CDCl_3_): *δ* = 7.55 (2H, d, *J* 8.0 Hz, H-2), 7.28 (1H, br. s, NH), 7.23 – 7.13 (7H, m, H-3, H-2’’’, H-3’’’, H-4’’’), 6.79 (2H, d, *J* 8.4 Hz, H-2’’), 6.56 (2H, d, *J* 8.4 Hz, H-3’’), 3.70 (3H, s, OCH_3_), 2.58 – 2.52 (1H, m, C*H*(CH_3_)_2_), 2.50 (2H, q, *J* 7.8 Hz, C*H*_2_CH_3_), 1.29 (6H, d, *J* 6.8 Hz, CH(C*H*_3_)_2_), 0.947 (3H, t, *J* 6.8 Hz, CH_2_C*H*_3_); ^13^C NMR (CDCl_3_): *δ* = 175.7 (CO), 158.4 (C-4’’), 143.3 (C-1’’’), 142.3 (C-2’), 140.6 (C-4), 138.6 (C-1’), 137.3 (C-1), 136.2 (C-1’’), 132.4 (C-2’’), 130.7 (C-3), 130.3 and 128.4 (C-2’’’ or C-3’’’), 126.6 (C-4’’’), 120.3 (C-2), 113.6 (C-3’’), 55.63 (OCH_3_), 37.26 (*C*H(CH_3_)_2_), 29.59 (*C*H_2_CH_3_), 20.12 (CH(*C*H_3_)_2_), 13.97 (CH_2_*C*H_3_).

**Compound 9: (*Z*)-*N*-(4-(1,2-diphenylbut-1-en-1-yl)phenyl)isobutyramide**

^1^H NMR (CDCl_3_): *δ* = 7.38 – 7.35 (2H, m, H-2’’), 7.31 – 7.25 (3H, m, H-3’’ and H-4’’), 7.20 (2H, d, *J* 8.4 Hz, H-2), 7.19 (2H, d, *J* 7.2 Hz, H-2’’’), 7.15 – 7.14 (3H, m, H-3’’’ and H-4’’’), 7.05 (1H, br. s, NH), 6.84 (2H, d, *J* 8.4 Hz, H-3), 2.49 (2H, q, *J* 7.6 Hz, C*H*_2_CH_3_), 2.47 – 2.38 (1H, m, C*H*(CH_3_)_2_), 1.21 (6H, d, *J* 7.2 Hz, CH(C*H*_3_)_2_), 0.956 (3H, t, *J* 7.6 Hz, CH_2_C*H*_3_); ^13^C NMR (CDCl_3_): *δ* = 175.3 (CO), 144.1 (C-1’’), 143.0 (C-2’), 142.8 (C-1’’’), 139.7 (C-4), 139.0 (C-1’), 136.4 (C-1), 131.9 (C-3), 130.3 (C-3’’’), 130.1 (C-3’’), 128.7 (C-2’’), 128.5 (C-2’’’), 127.2 (C-4’’), 126.8 (C-4’’’), 119.4 (C-2), 37.20 (*C*H(CH_3_)_2_), 29.61 (*C*H_2_CH_3_), 20.07 (CH(*C*H_3_)_2_), 13.94 (CH_2_*C*H_3_).

**Compound 10: (*E*)-*N*-(4-(1,2-diphenylbut-1-en-1-yl)phenyl)isobutyramide**

^1^H NMR (acetone-d_6_): *δ* = 9.11 (1H, br. s, NH), 7.72 (2H, d, *J* 8.8 Hz, H-2), 7.38 (2H, t, *J* 8.8 Hz, H-3’’), 7.30 (1H, t, *J* 8.8 Hz, H-4’’), 7.20 (2H, d, *J* 8.8 Hz, H-3), 7.03 – 6.99 (3H, m, H-3’’’ and H-4’’’), 6.93 (2H, d, *J* 8.4 Hz, H-2’’’), 6.83 (2H, d, *J* 8.8 Hz, H-2’’), 2.68 – 2.61 (1H, m, C*H*(CH_3_)_2_), 2.53 (2H, q, *J* 7.6 Hz, C*H*_2_CH_3_), 1.19 (6H, d, *J* 7.2 Hz, CH(C*H*_3_)_2_), 0.948 (3H, t, *J* 7.6 Hz, CH_2_C*H*_3_); ^13^C NMR (acetone-d_6_): *δ* = 175.0 (CO), 143.3 (C-1’’), 142.3 (C-2’), 138.7 (C-1’’’), 138.4 and 138.3 (C1 and/or C-4), 137.8 (C-1’), 130.8 (C-2’’), 130.6 (C-2’’’), 129.5 (C-3), 128.2 (C-3’’), 127.8 (C-3’’’), 126.6 (C-4’’), 125.7 (C-4’’’), 118.8 (C-2), 35.72 (*C*H(CH_3_)_2_), 28.82 (*C*H_2_CH_3_ from HSQC), 19.02 (CH(*C*H_3_)_2_), 12.89 (CH_2_*C*H_3_).

**Compound 11: (*E*)-*N*-(4-(2-(4-(benzyloxy)phenyl)-1-phenylbut-1-en-1-yl)phenyl) isobutyramide**

^1^H NMR (acetone-d_6_): *δ* = 8.89 (1H, br. s, NH), 7.48 (2H, br. d, *J* 6.8 Hz, *o*-Ph*H*CH_2_), 7.43 – 7.30 (8H, m, *m*-Ph*H*CH_2_, *p*-Ph*H*CH_2_, H-3’’’, H-4’’’, H-2), 7.27 (2H, d, *J* 8.0 Hz, H-3), 7.13 (2H, d, *J* 8.8 Hz, H-2’’), 6.88 (2H, d, *J* 8.8 Hz, H-3’’), 6.84 (2H, d, *J* 8.8 Hz, H-2’’’), 5.07 (2H, s, CH_2_O), 2.60 – 2.51 (1H, m, C*H*(CH_3_)_2_), 2.46 (2H, q, *J* 7.2 Hz, C*H*_2_CH_3_), 1.13 (6H, d, *J* 6.8 Hz, CH(C*H*_3_)_2_), 0.933 (3H, t, *J* 7.6 Hz, CH_2_C*H*_3_); ^13^C NMR (acetone-d_6_): *δ* = 174.8 (CO), 157.4 (C-4’’), 143.9 (C-4), 141.1 (C-2’), 138.2 (C-1 and C-1’), 137.5 (C1’’’ and Ph*C*-CH_2_O), 134.6 (C-1’’), 130.9 (C-2’’’), 130.8 (C-2’’), 129.3 (C-3), 128.4 (Ph*C*-para), 128.1 (C-3’’’), 127.7 and 127.6 (Ph*C*-ortho and/or Ph*C*-meta), 126.5 (C-4’’’), 118.2 (C-2), 114.2 (C-3’’), 69.52 (CH_2_O), 35.70 (*C*H(CH_3_)_2_), 28.44 (*C*H_2_CH_3_ from HSQC), 18.98 (CH(*C*H_3_)_2_), 12.98 (CH_2_*C*H_3_).

**Compound 12: (*Z*)-*N*-(4-(2-(4-(benzyloxy)phenyl)-1-phenylbut-1-en-1-yl)phenyl) isobutyramide**

^1^H NMR (acetone-d_6_): *δ* = 9.08 (1H, br. s, NH), 7.71 (2H, d, *J* 8.8 Hz, H-2), 7.47 (2H, br. d, *J* 6.8 Hz, *o*-Ph*H*CH_2_), 7.39 (2H, t, *J* 6.4 Hz, *m*-Ph*H*CH_2_), 7.28 (1H, t, *J* 6.8 Hz, *p*-Ph*H*CH_2_), 7.18 (2H, d, *J* 8.8 Hz, H-3), 7.09 (2H, d, *J* 8.8 Hz, H-2’’), 7.05 – 6.98 (3H, m, H-3’’’ and H-4’’’), 6.94 (2H, dd, *J* 8.4 Hz *J* 1.6 Hz, H-2’’’), 6.84 (2H, d, *J* 8.8 Hz, H-3’’), 5.05 (2H, s, CH_2_O), 2.69 – 2.59 (1H, m, C*H*(CH_3_)_2_), 2.50 (2H, q, *J* 7.6 Hz, C*H*_2_CH_3_), 1.19 (6H, d, *J* 6.8 Hz, CH(C*H*_3_)_2_), 0.950 (3H, t, *J* 7.6 Hz, CH_2_C*H*_3_); ^13^C NMR (acetone-d_6_): *δ* = 175.7 (CO), 158.0 (C-4’’), 144.2 (C-1’’’), 142.1 (C-2’), 139.2 (C-4), 139.0 (C-1), 138.8 (C-1’), 138.2 (Ph*C*-CH_2_O), 135.2 (C-1’’), 131.4 (C-2’’), 131.3 (C-2’’’), 130.2 (C-3), 130.0 (Ph*C*-para), 129.0 (Ph*C*-meta), 128.4 (Ph*C*-ortho), 128.0 (C-3’’’), 126.3 (C-4’’’), 120.0 (C-2), 114.8 (C-3’’), 70.15 (CH_2_O), 36.44 (*C*H(CH_3_)_2_), 29.24 (*C*H_2_CH_3_ from HSQC), 19.72 (CH(*C*H_3_)_2_), 13.71 (CH_2_*C*H_3_).

**Compound 13: (*E*)-*N*-(4-(2-(2,4-dichlorophenyl)-1-(4-hydroxyphenyl)but-1-en-1-yl)phenyl) isobutyramide**

^1^H NMR (acetone-d_6_): *δ* = 8.90 (1H, br. s, NH), 8.43 (1H, br. s, OH), 7.40 – 7.38 (3H, m, H-2 and H-5’’), 7.28 (1H, d, *J* 8.0 Hz, H-6’’), 7.25 (1H, d, *J* 2.0 Hz, H-3’’), 7.14 (2H, d, *J* 8.4 Hz, H-2’’’), 6.89 (2H, d, *J* 8.8 Hz, H-3), 6.88 (2H, d, *J* 8.4 Hz, H-3’’’), 2.58 – 2.52 (1H, m, C*H*(CH_3_)_2_), 2.49 (2H, q, *J* 7.6 Hz, C*H*_2_CH_3_), 1.11 (6H, d, *J* 6.8 Hz, CH(C*H*_3_)_2_), 0.953 (3H, t, *J* 7.6 Hz, CH_2_C*H*_3_); ^13^C NMR (acetone-d_6_): *δ* = 175.6 (CO), 157.2 (C-4’’’), 141.9 (C-1’), 140.8 (C-1’’), 138.6 (C-1), 138.3 (C-2’ and C-4), 137.6 (C-1’’’), 134.7 (C-2’’ or C-4’’), 134.2 (C-6’’), 132.9 (C-2’’ or C-4’’), 131.0 (C-2’’’), 130.4 (C-3), 129.4 (C-5’’), 127.4 (C-3’’), 118.7 (C-2), 115.6 (C-3’’’), 36.37 (*C*H(CH_3_)_2_), 28.45 (*C*H_2_CH_3_), 19.63 (CH(*C*H_3_)_2_), 13.04 (CH_2_*C*H_3_).

**Compound 14: (*Z*)-*N*-(4-(2-(2,4-dichlorophenyl)-1-(4-hydroxyphenyl)but-1-en-1-yl)phenyl) isobutyramide**

^1^H NMR (acetone-d_6_): *δ* = 9.13 (1H, br. s, NH), 8.18 (1H, br. s, OH), 7.72 (2H, d, *J* 8.4 Hz, H-2), 7.40 – 7.38 (1H, m, H-5’’), 7.27 (1H, d, *J* 8.0 Hz, H-6’’), 7.26 (1H, d, *J* 2.0 Hz, H-3’’), 7.22 (2H, d, *J* 8.8 Hz, H-3), 6.80 (2H, d, *J* 8.8 Hz, H-2’’’), 6.54 (2H, d, *J* 8.8 Hz, H-3’’’), 2.70 – 2.60 (1H, m, C*H*(CH_3_)_2_), 2.47 (2H, q, *J* 7.6 Hz, C*H*_2_CH_3_), 1.18 (6H, d, *J* 6.8 Hz, CH(C*H*_3_)_2_), 0.947 (3H, t, *J* 7.2 Hz, CH_2_C*H*_3_); ^13^C NMR (acetone-d_6_): *δ* = 175.7 (CO), 156.6 (C-4’’’), 141.9 (C-1’), 140.9 (C-1’’), 139.3 (C-1), 138.1 (C-4), 137.4 (C-2’), 134.7 (C-2’’ or C-4’’), 134.4 (C-1’’’), 134.2 (C-6’’), 132.8 (C-2’’ or C-4’’), 131.3 (C-2’’’), 130.1 (C-3), 129.4 (C-5’’), 127.4 (C-3’’), 119.5 (C-2), 114.9 (C-3’’’), 36.40 (*C*H(CH_3_)_2_), 28.40 (*C*H_2_CH_3_), 19.67 (CH(*C*H_3_)_2_), 13.03 (CH_2_*C*H_3_).

**Compound 15: (*E*)-*N*-(4-(2-(4-(benzyloxy)phenyl)-1-(4-methoxyphenyl)but-1-en-1-yl)phenyl) isobutyramide**

^1^H NMR (acetone-d_6_): *δ* = 8.87 (1H, br. s, NH), 7.48 (2H, br. d, *J* 6.8 Hz, *o*-Ph*H*CH_2_), 7.48 – 7.38 (4H, m, H-2 and *m*-Ph*H*CH_2_), 7.34 (1H, t, *J* 7.2 Hz, *p*-Ph*H*CH_2_), 7.17 (2H, d, *J* 8.8 Hz, H-2’’’), 7.11 (2H, d, *J* 8.8 Hz, H-2’’), 6.95 (2H, d, *J* 8.8 Hz, H-3’’’), 6.86 (2H, d, *J* 8.8 Hz, H-3’’), 6.83 (2H, d, *J* 8.8 Hz, H-3), 5.06 (2H, s, CH_2_O), 3.83 (3H, s, CH_3_O), 2.60 – 2.53 (1H, m, C*H*(CH_3_)_2_), 2.49 (2H, q, *J* 7.6 Hz, C*H*_2_CH_3_), 1.13 (6H, d, *J* 7.2 Hz, CH(C*H*_3_)_2_), 0.940 (3H, t, *J* 7.6 Hz, CH_2_C*H*_3_); ^13^C NMR (acetone-d_6_): *δ* = 174.8 (CO), 158.6 (C-4’’’), 157.3 (C-4’’), 140.8 (C-2’), 138.5 (C-4), 137.8 (C-1’), 137.6 (C-1’’), 137.4 (Ph*C*-CH_2_O), 136.1 (C-1’’’), 134.8 (C-1), 130.9 (C-3), 130.8 (C-2’’), 130.4 (C-2’’’), 128.4 (Ph*C*-meta), 127.7 (Ph*C*-para), 127.6 (Ph*C*-ortho), 118.1 (C-2), 114.2 (C-3’’), 113.5 (C-3’’’), 69.51 (CH_2_O), 54.60 (OCH_3_), 35.71 (*C*H(CH_3_)_2_), 28.82 (*C*H_2_CH_3_ from HSQC), 18.99 (CH(*C*H_3_)_2_), 13.03 (CH_2_*C*H_3_).

**Compound 16: (*Z*)-*N*-(4-(2-(4-(benzyloxy)phenyl)-1-(4-methoxyphenyl)but-1-en-1-yl)phenyl) isobutyramide**

^1^H NMR (acetone-d_6_): *δ* = 9.08 (1H, br. s, NH), 7.70 (2H, d, *J* 8.4 Hz, H-2), 7.48 (2H, d, *J* 7.2 Hz, *o*-Ph*H*CH_2_), 7.40 (2H, t, *J* 7.2 Hz, *m*-Ph*H*CH_2_), 7.34 (1H, t, *J* 7.2 Hz, *p*-Ph*H*CH_2_), 7.16 (2H, d, *J* 8.4 Hz, H-3), 7.10 (2H, d, *J* 8.8 Hz, H-2’’), 6.86 (2H, d, *J* 8.8 Hz, H-3’’), 6.83 (2H, d, *J* 8.8 Hz, H-2’’’), 6.61 (2H, d, *J* 8.8 Hz, H-3’’’), 5.07 (2H, s, CH_2_O), 3.69 (3H, s, CH_3_O), 2.68 – 2.61 (1H, m, C*H*(CH_3_)_2_), 2.48 (2H, q, *J* 7.2 Hz, C*H*_2_CH_3_), 1.19 (6H, d, *J* 6.8 Hz, CH(C*H*_3_)_2_), 0.935 (3H, t, *J* 7.2 Hz, CH_2_C*H*_3_); ^13^C NMR (acetone-d_6_): *δ* = 175.6 (CO), 158.5 (C-4’’’), 158.0 (C-4’’), 141.3 (C-2’), 139.6 (C-4), 138.9 (C-1), 138.6 (C-1’), 138.3 (Ph*C*-CH_2_O), 136.5 (C-1’’’), 135.7 (C-1’’), 132.3 (C-2’’’), 131.4 (C-2’’), 130.1 (C-3), 128.9 (Ph*C*-meta), 128.2 (Ph*C*-para), 128.1 (Ph*C*-ortho), 119.7 (C-2), 115.0 (C-3’’), 113.5 (C-3’’’), 70.37 (CH_2_O), 55.08 (OCH_3_), 36.42 (*C*H(CH_3_)_2_), 29.42 (*C*H_2_CH_3_ from HSQC), 19.57 (CH(*C*H_3_)_2_), 13.53 (CH_2_*C*H_3_).

**Compound 17:** **(*Z*)-*N*-(4-(2-(4-hydroxyphenyl)-1-(4-methoxyphenyl)but-1-en-1-yl)phenyl) isobutyramide**

^1^H NMR (acetone-d_6_): *δ* = 9.13 (1H, br. s, NH), 8.25 (1H, br. s, OH), 7.70 (2H, d, *J* 8.4 Hz, H-2), 7.15 (2H, d, *J* 8.4 Hz, H-3), 6.99 (2H, d, *J* 8.8 Hz, H-2’’), 6.82 (2H, d, *J* 8.8 Hz, H-2’’’), 6.68 (2H, d, *J* 8.8 Hz, H-3’’), 6.61 (2H, d, *J* 8.8 Hz, H-3’’’), 3.68 (3H, s, CH_3_O), 2.68 – 2.61 (1H, m, C*H*(CH_3_)_2_), 2.46 (2H, q, *J* 7.6 Hz, C*H*_2_CH_3_), 1.19 (6H, d, *J* 6.8 Hz, CH(C*H*_3_)_2_), 0.932 (3H, t, *J* 7.6 Hz, CH_2_C*H*_3_); ^13^C NMR (acetone-d_6_): *δ* = 175.6 (CO), 158.5 (C-4’’’), 156.4 (C-4’’), 141.7 (C-2’), 139.8 (C-4), 138.8 (C-1), 137.8 (C-1’), 136.7 (C-1’’’), 134.2 (C-1’’), 132.2 (C-2’’’), 131.3 (C-2’’), 130.1 (C-3), 119.9 (C-2), 115.5 (C-3’’), 113.5 (C-3’’’), 55.09 (OCH_3_), 36.42 (*C*H(CH_3_)_2_), 29.08 (*C*H_2_CH_3_ from HSQC), 19.54 (CH(*C*H_3_)_2_), 13.47 (CH_2_*C*H_3_).

**Compound 18: (*E*)-*N*-(4-(2-(4-hydroxyphenyl)-1-(4-methoxyphenyl)but-1-en-1-yl)phenyl) isobutyramide**

^1^H NMR (acetone-d_6_): *δ* = 8.86 (1H, br. s, NH), 8.16 (1H, br. s, OH), 7.37 (2H, d, *J* 8.4 Hz, H-2), 7.16 (2H, d, *J* 8.4 Hz, H-2’’’), 7.00 (2H, d, *J* 8.4 Hz, H-2’’), 6.94 (2H, d, *J* 8.8 Hz, H-3’’’), 6.82 (2H, d, *J* 8.8 Hz, H-3), 6.68 (2H, d, *J* 8.8 Hz, H-3’’), 3.83 (3H, s, CH_3_O), 2.59 – 2.52 (1H, m, C*H*(CH_3_)_2_), 2.47 (2H, q, *J* 7.2 Hz, C*H*_2_CH_3_), 1.12 (6H, d, *J* 6.8 Hz, CH(C*H*_3_)_2_), 0.940 (3H, t, *J* 7.2 Hz, CH_2_C*H*_3_); ^13^C NMR (acetone-d_6_): *δ* = 174.8 (CO), 158.5 (C-4’’’), 155.7 (C-4’’), 141.1 (C-2’), 138.7 (C-4), 137.3 (C-1 and C-1’), 136.2 (C-1’’’), 133.3 (C-1’’), 130.9 (C-3), 130.7 (C-2’’), 130.4 (C-2’’’), 118.1 (C-2), 114.8 (C-3’’), 113.4 (C-3’’’), 54.59 (OCH_3_), 35.70 (*C*H(CH_3_)_2_), 28.83 (*C*H_2_CH_3_ from HSQC), 18.99 (CH(*C*H_3_)_2_), 13.06 (CH_2_*C*H_3_).

**Compound 19: (*E*)-*N*-(4-(1-(4-hydroxyphenyl)-2-phenylbut-1-en-yl)phenyl)isobutyramide**

^1^H NMR (CDCl_3_): *δ* = 7.28 (1H, br. s, NH), 7.20 – 7.11 (9H, m, H-2, H-2’’, H-2’’’, H-3’’’ and H-4’’’), 6.83 (4H, br. d, *J* 7.6 Hz, H-3 and H-3’’), 2.53 – 2.45 (3H, m, C*H*(CH_3_)_2_ and C*H*_2_CH_3_), 1.21 (6H, d, *J* 6.8 Hz, CH(C*H*_3_)_2_), 0.951 (3H, t, *J* 7.6 Hz, CH_2_C*H*_3_); ^13^C NMR (CDCl_3_): *δ* = 175.4 (CO), 155.2 (C-4’’), 143.2 (C-1’’’), 142.3 (C-2’), 140.6 (C-4), 138.5 (C-1), 136.7 (C-1’), 136.3 (C-1’’), 131.9 and 131.4 (C-3 and/or C-2’’), 130.3 (C-2’’’), 128.4 (C-3’’’), 126.6 (C-4’’’), 119.6 (C-2), 115.8 (C-3’’), 37.26 (*C*H(CH_3_)_2_), 29.62 (*C*H_2_CH_3_), 20.06 (CH(*C*H_3_)_2_), 13.95 (CH_2_*C*H_3_).

**Compound 20: (*Z*)-*N*-(4-(1-(4-hydroxyphenyl)-2-phenylbut-1-en-yl)phenyl)isobutyramide**

^1^H NMR (CDCl_3_): *δ* = 7.53 (2H, d, *J* 8.0 Hz, H-2), 7.29 (1H, br. s, NH), 7.21 (2H, d, *J* 8.4 Hz, H-3), 7.18 (2H, d, *J* 8.0 Hz, H-3’’’), 7.14 – 7.11 (3H, m, H-2’’’and H-4’’’), 6.72 (2H, d, *J* 8.4 Hz, H-2’’), 6.50 (2H, d, *J* 8.4 Hz, H-3’’), 2.59 – 2.53 (1H, m, C*H*(CH_3_)_2_), 2.49 (2H, q, *J* 7.2 Hz, C*H*_2_CH_3_), 1.29 (6H, d, *J* 6.4 Hz, CH(C*H*_3_)_2_), 0.942 (3H, t, *J* 6.8 Hz, CH_2_C*H*_3_); ^13^C NMR (CDCl_3_): *δ* = 175.5 (CO), 153.8 (C-4’’), 142.4 (C-1’’’), 141.5 (C-2’), 140.0 (C-4), 137.6 (C-1), 136.4 (C-1’), 135.4 (C-1’’), 132.1 (C-2’’), 130.2 (C-3), 129.7 (C-2’’’), 127.9 (C-3’’’), 126.0 (C-4’’’), 119.6 (C-2), 114.4 (C-3’’), 36.79 (*C*H(CH_3_)_2_), 29.04 (*C*H_2_CH_3_), 19.67 (CH(*C*H_3_)_2_), 13.62 (CH_2_*C*H_3_).

**Compound 21: (*E*)-*N*-(4-(1’,2’-bis(4’’,4’’’-(benzyloxy)phenyl)but-1’-enyl)phenyl)isobutyramide**

^1^H NMR (CDCl_3_, 600 MHz): *δ* = 7.47 – 7.35 (10H, m, Ph*H* and Ph*Η*’), 7.20 (2H, d, *J* 8.4 Hz, H-2), 7.14 (2H, d, *J* 8.4 Hz, H-2’’), 7.03 (2H, d, *J* 8.4 Hz, H-2’’’), 7.00 (1Η, s, N*H*), 6.95 (2H, d, *J* 8.4 Hz, H-3’’), 6.83 (2H, d, *J* 8.4 Hz, H-3), 6.79 (2H, d, *J* 8.4 Hz, H-3’’’), 5.07 (2H, s, C4’’-OC*H*_2_), 5.00 (2H, s, C4’’’-OC*H*_2_), 2.49 (2H, q, *J* 7.8 Hz, C*H*_2_CH_3_), 2.46 – 2.41 (1H, m, C*H*(CH_3_)_2_), 1.21 (6H, d, *J* 6.6 Hz, CH(C*H*_3_)_2_), 0.945 (3H, t, *J* 7.8 Hz, CH_2_C*H*_3_); ^13^C NMR (CDCl_3_, 50 MHz): *δ* = 175.0 (CO), 157.6 (C-4’’), 157.2 (C-4’’’), 141.2 (C-2’), 140.0 (C-4), 137.3 (C-1), 137.2 (Ph*C*CH_2_O and Ph*C’*CH_2_O), 136.5 (C-1’), 136.0 (C-1’’), 135.6 (C-1’’’), 131.6 (C-3), 130.8 (C-2’’’), 130.7 (C-2’’), 128.6 (Ph*C’*-ortho and Ph*C*-ortho), 128.0 (Ph*C’*-para and Ph*C*-para), 127.6 (Ph*C’*-meta and Ph*C*-meta), 118.6 (C-2), 114.4 (C-3’’), 113.4 (C-3’’’), 70.1 (C4’’-O*C*H_2_), 70.0 (C4’’’-O*C*H_2_), 36.7 (*C*H(CH_3_)_2_), 28.7 (*C*H_2_CH_3_), 19.7 (CH(*C*H_3_)_2_), 13.7 (CH_2_*C*H_3_).

**Compound 22: (*Z*)-*N*-(4-(1’,2’-bis(4’’,4’’’-(benzyloxy)phenyl)but-1’-enyl)phenyl)isobutyramide**

^1^H NMR (CDCl_3_, 600 MHz): *δ* = 7.51 (2H, d, *J* 8.4 Hz, H-2), 7.46 – 7.29 (10H, m, Ph*H* and Ph*Η*’), 7.19 (2H, d, *J* 8.4 Hz, H-3), 7.16 (1H, s, N*H*), 7.04 (2H, d, *J* 8.4 Hz, H-2’’’), 6.80 (2H, d, *J* 8.4 Hz, H-3’’’), 6.78 (2H, d, *J* 8.4 Hz, H-2’’), 6.64 (2H, d, *J* 8.4 Hz, H-3’’), 5.01 (2H, s, C4’’’-OC*H*_2_), 4.93 (2H, s, C4’’-OC*H*_2_), 2.53 – 2.48 (1H, m, C*H*(CH_3_)_2_), 2.44 (2H, q, *J* 7.8 Hz, C*H*_2_CH_3_), 1.27 (6H, d, *J* 6.6 Hz, CH(C*H*_3_)_2_), 0.935 (3H, t, *J* 7.8 Hz, CH_2_C*H*_3_); ^13^C NMR (CDCl_3_, 50 MHz): *δ* = 175.2 (CO), 157.1 (C-4’’’), 156.7 (C-4’’), 141.0 (C-2’), 139.9 (C-4), 137.2 (Ph*C*CH_2_O and Ph*C’*CH_2_O), 136.4 (C-1’), 136.0 (C-1’’), 134.9 ( C-1’’’), 132.0 (C-2’’), 130.8 (C-1), 130.7 (C-2’’’), 130.1 (C-3), 128.5 (Ph*C’*-ortho and Ph*C*-ortho), 127.9 (Ph*C’*-para and Ph*C*-para), 127.5 (Ph*C’*-meta and Ph*C*-meta), 119.4 (C-2), 114.3 (C-3’’’), 113.7 (C-3’’), 69.9 (C4’’’-O*C*H_2_), 69.8 (C4’’-O*C*H_2_), 36.8 (*C*H(CH_3_)_2_), 29.7 (*C*H_2_CH_3_), 19.6 (CH(*C*H_3_)_2_), 13.7 (CH_2_*C*H_3_).
